# An integrative analysis of small molecule transcriptional responses in the human malaria parasite *Plasmodium falciparum*

**DOI:** 10.1186/s12864-015-2165-1

**Published:** 2015-12-04

**Authors:** Geoffrey H. Siwo, Roger S. Smith, Asako Tan, Katrina A. Button-Simons, Lisa A. Checkley, Michael T. Ferdig

**Affiliations:** Eck Institute for Global Health, Department of Biological Sciences, University of Notre Dame, Notre Dame, IN 46556 USA; Current Address: IBM TJ Watson Research Center, Yorktown Heights, NY 10598 USA; Current Address: IBM Research-Africa, South Africa Lab, Sandton, Johannesburg, 2196 South Africa; Current Address: Memorial Sloan Kettering Cancer Center, New York, NY 10065 USA; Epicenter, Madison, WI 53719 USA

## Abstract

**Background:**

Transcriptional responses to small molecules can provide insights into drug mode of action (MOA). The capacity of the human malaria parasite, *Plasmodium falciparum*, to respond specifically to transcriptional perturbations has been unclear based on past approaches. Here, we present the most extensive profiling to date of the parasite’s transcriptional responsiveness to thirty-one chemically and functionally diverse small molecules.

**Methods:**

We exposed two laboratory strains of the human malaria parasite *P. falciparum* to brief treatments of thirty-one chemically and functionally diverse small molecules associated with biological effects across multiple pathways based on various levels of evidence. We investigated the impact of chemical composition and MOA on gene expression similarities that arise between perturbations by various compounds. To determine the target biological pathways for each small molecule, we developed a novel framework for encoding small molecule effects on a spectra of biological processes or GO functions that are enriched in the differentially expressed genes of a given small molecule perturbation.

**Results:**

We find that small molecules associated with similar transcriptional responses contain similar chemical features, and/ or have a shared MOA. The approach also revealed complex relationships between drugs and biological pathways that are missed by most exisiting approaches. For example, the approach was able to partition small molecule responses into drug-specific effects versus non-specific effects.

**Conclusions:**

Our work provides a new framework for linking transcriptional responses to drug MOA in *P. falciparum* and can be generalized for the same purpose in other organisms.

**Electronic supplementary material:**

The online version of this article (doi:10.1186/s12864-015-2165-1) contains supplementary material, which is available to authorized users.

## Background

Malaria continues to take a large toll on the health and economies of some of the world’s poorest nations. Drugs remain the primary option for dealing with malaria infection although there are promising clinical trials that may pave the way for the use of vaccines against the disease [1]. In spite of enormous progress in the fight against the disease, the emergence of drug resistance to artemisinin, the only anti-malarial drug for which clinical resistance is not yet widespread, threatens to reverse the gains [2, 3]. There is an urgent need to fast-track the development of new anti-malarials. Fortunately, high-throughput and phenotypic screens have provided several potential drug leads. Through public and private efforts, nearly six million compounds have been screened leading to the identification of thousands of active compounds [4–6]. The Malaria Box, an open access “pharmacological test kit” has been made freely available to malaria researchers in an effort to spur antimalarial dug development [6]. The malaria drug development pipeline now contains over a dozen new drugs and several new combinations of approved drugs are in various stages of pre-clinical and clinical trials [7]. Approximately half of these new drugs have unknown mechanisms. An understanding of the mode of action (MOA) of these compounds would help prioritize and optimize them while also highlighting potential resistance mechanisms. This could help mitigate the high rate of failure in the drug development pipeline or rapid emergence of drug resistance. Furthermore, even compounds with no potential to be developed into useful therapeutics are valuable as tools for probing the parasite’s biology [6].

Transcriptional profiling of cells exposed to small molecules has been successfully demonstrated as useful in understanding drug MOA especially in human cell lines. For instance, the Connectivity Map (CMap) [8, 9], a database of gene expression profiles from cancer cell lines exposed to multiple drugs of known MOA has been used to successfully predict the MOA of new drugs [10, 11]. However, chemical perturbation of *P. falciparum*, which has been widely described as transcriptionally hard-wired [12–14], is thought to provoke little in the way of a specific response. For example, treatment of the parasite with lethal anti-folate drugs for up to 24 hrs did not reproducibly induce up-regulation of genes in the folate pathway or elsewhere in the genome [12]. Exposure to chloroquine (CQ) was reported to have no effect on the transcript levels of genes presumed to be involved in its MOA [13, 14]. However, in other studies, perturbation of the parasite with a polyamine synthesis inhibitor, diflouromethylornithine- DFMO [15], febrile temperatures [16], artesunate [17, 18] and an inhibitor of sphingomyelin synthase [19] were found to provoke transcriptional changes in the expected target biological pathways. These reports suggest that the reported lack of responsiveness to CQ and anti-folate drugs cannot be generalized to all perturbations. In an earlier large-scale perturbation of malaria parasites with 20 drugs [20], 59 % of genes responded transcriptionally to at least one. Some drugs, including CQ, quinine and colcichine affected fewer than 50 genes, while drugs like apicidin, trichostatin A and staurosporine affected more than 250 genes and, importantly, even for drugs that only modestly affected gene expression, the effects were highly reproducible to those in other experiments within the same study [20]. The ability of the parasite to respond in a reproducible way implies that the response is physiologically relevant and coordinated. Except for the study by Hu et al. [20], transcriptional studies in the parasite have focused on only one drug at a time, making it difficult to reach general conclusions about the parasite’s capacity to mount specific transcriptional responses [12, 13, 15]. We expand on these previous reports to investigate the extent to which transcriptional responses in *P. falciparum* can indicate precise small molecule targets and/or broader biological effects. To do this, we devised a 6 step approach that involves: i) performing perturbations with 31 chemically and functionally diverse drugs; ii) minimizing biological variation among samples by leveraging the multiplex exon array developed in our lab; iii) generating perturbations in two genetically and phenotypically distinct lab clones; iv) exposing parasites only briefly (2 hrs) to small molecules to minimize secondary effects; v) minimizing non-specific perturbation effects by normalizing transcript levels relative to all other perturbations rather than to untreated controls as is typically done, and, vi) leveraging multiple independent datasets to cross-validate that transcriptional responses reflect biologically meaningful small molecule relationships.

## Results

### Overview of the study

We compiled a set of 31 chemically and functionally diverse small molecules associated with inhibition of biological processes involving a wide range of cellular compartments: the cell membrane (cerulenin, *dl*-threo-1-phenyl-2-palmitoylamino-3-morpholino-1-propanol (PPMP), dideoxyadenosine, fenofibrate), cytoplasm (geldanamycin, methotrexate, epoxomicin, vincristine, 5-fluorouracil), nucleus (methyl methane sulfonate, olomucine, apicidin, JQ1, curcumin), digestive vacuole (chloroquine, tafenoquine, nelfinavir, E64), mitochondria (proguanil, atovaquone, z-Val-Asp-fluoromethylketone, chloramphenicol, doxycycline, rapamycin) and apicoplast (doxycycline, chloramphenicol, cerulenin, PPMP). Six compounds (artemisinin and five novel compounds- SJ194935, SJ119930, SJ140722, SJ292024 and SJ77572) lacked adequate information to be associated with any biological process or cellular compartment. Detailed information for all compounds is provided in Table 1 alongside their PubChem identification ids (CID) for retrieving their chemical structures and additional information. The association between each of the compounds and a given biological process in *P. falciparum* is supported by various levels of evidence extracted from literature sources and the BRENDA enzyme database [21]. Criteria utilized in selecting the small molecules include parasite growth inhibition (IC_50_), enzyme or functional assays, protein structures with co-crystalized compound in the protein data bank (PDB) and molecular docking simulations (Table 1).

**Table 1:**
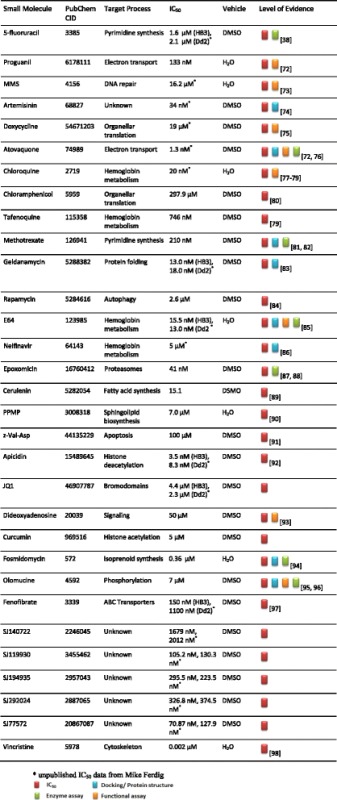
Summary of small molecule perturbations

Synchronized cultures of two laboratory clones, Dd2 and HB3, were exposed for 2 h to each compound at a single developmental stage (24 h trophozoites) at a concentration (IC_50_) obtained from literature sources or determined by our laboratory (Table 1). Genome-wide transcript abundances were determined for each drug perturbation by microarrays.

Previous studies in *P. falciparum* noted only very subtle transcriptional responses, largely attributed to generalized stress responses [12, 13, 20]. Therefore, we undertook additional data processing steps of RMA-normalized (see Methods) signal intensity data. First, for each perturbation, we averaged the signal intensity for each gene across two distinct lab clones (Dd2 and HB3), to enhance signal-to-noise ratio as demonstrated in the CMap project [10, 22]. We further validated that averaging gene expression data across two different strains in this way strengthens the identification of drug MOA (see Additional file 1: section D). Importantly, because the two clones have very different geographical and drug selection histories, they effectively serve as robustly independent replicates for each perturbation. Secondly, we computed a compound-specific response index for each gene by normalizing the gene’s average transcript level following perturbations in the two clones against its average level across all perturbations within the same experimental batch (Additional file 2) to increase signal-to-noise ratio [23]. This normalization procedure uses distinct biological replicates to help mitigate non-specific transcriptional responses associated with many perturbations as well as experimental batch effects. This approach differs significantly from previous studies [12, 13, 20] in *P. falciparum* that relied on normalization using untreated controls for single perturbations; in this case, widespread non-specific transcriptional responses can obscure perturbation-specific responses (additional evidence in support of this is in Additional file 1: section D).

### Global relationships in small molecule transcriptional responses relate to chemical structure

**Fig. 1:**
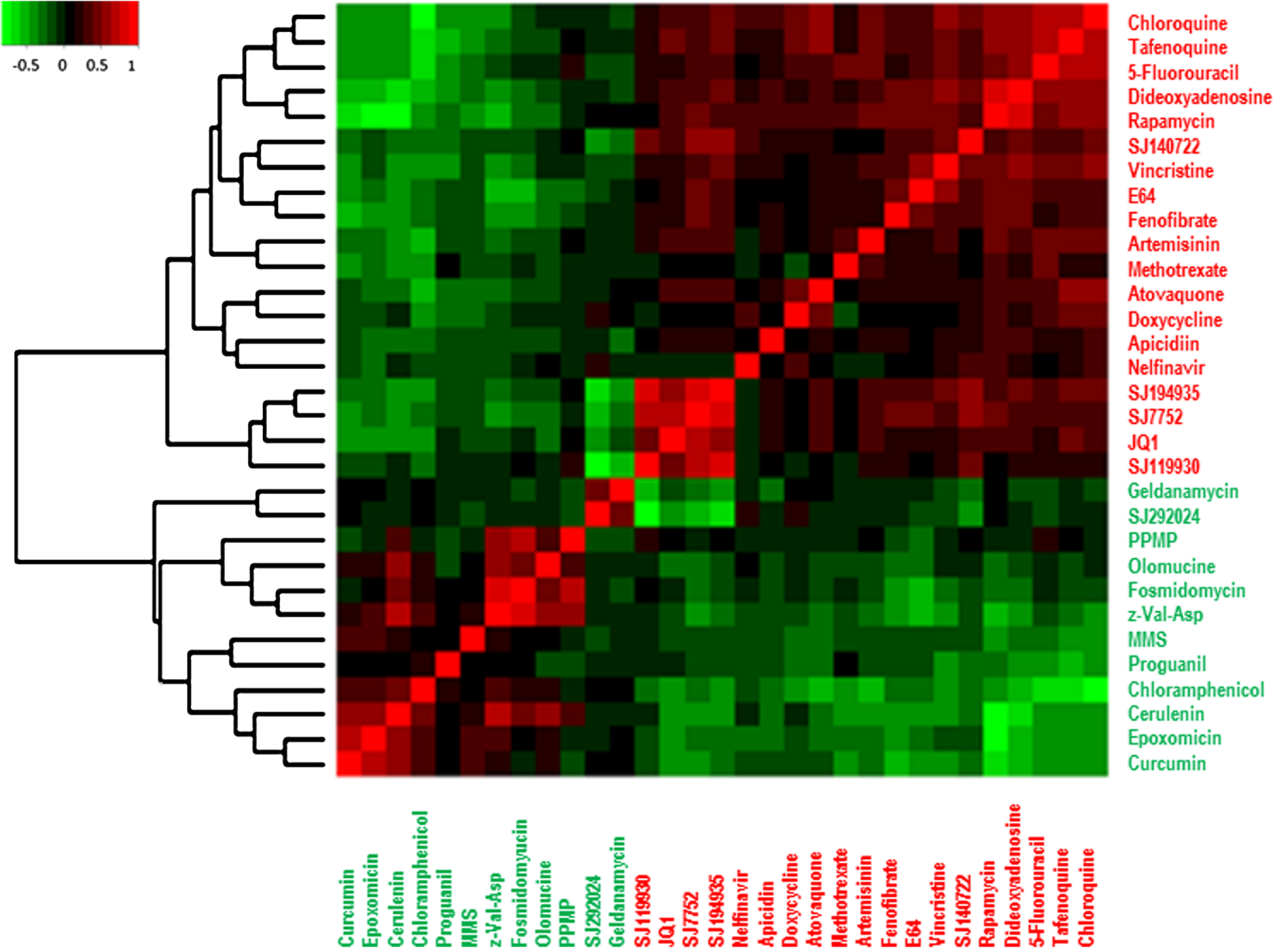
Hierarchical clustering of compounds based on their genome-wide response indices for each gene across two parasite samples. The respond index for each gene in a given perturbation is obtained as the ratio between the average transcript level of the gene following a 2 h exposure to the compound in two parasite samples and the average transcript level of the same gene across all perturbations performed in the same batch of experiments. The clustering of the compounds reveals two broad groupings (*Class I- red labels and Class II- green labels*) in which compounds within a group are positively correlated to each other but negatively correlated to compounds in a different group

To obtain a global view of transcriptional relationships between small molecules, we computed a correlation between each pair of compounds using the genome-wide response index (Fig. 1, Additional file 1 for data). The genome-wide response index for each compound is the response index for each gene following exposure to the compound. All 31 compounds clustered into only two broad groups (Fig. 1), hereafter referred to as Class I and II. Class I compounds are generally highly positively correlated to compounds within the class but are negatively correlated to those of Class II. Similarly, Class II compounds tend to be highly positively correlated to each other but negatively correlated to those in Class I (Fig. 1). These broad groupings have not been reported before, possibly because previous studies involved only one or two drug perturbations and the largest study to date involved 20 drugs [20].

The biological response of cells to small molecules is dependent on chemical structure. Small molecules with similar chemical components have similar biological effects as supported by structure activity relationships (SAR) studies and correlations between gene expression effects of drugs and their chemical structures [24–27]. To better understand the role of chemical structure on the observed dichotomy (Fig. 1), we hierarchically clustered the compounds based on their PubChem substructure fingerprints (Additional file 1: Figure S1; see Additional file 3 for the fingerprints). The PubChem substructure fingerprint clusters to some extent recapitulate the transcription profile clusters (Additional file 1: Figure S1). In particular, 8 of 9 (89 %) Class I compounds (based on transcriptional responses) also cluster together purely based on substructure fingerprints (hypergeometric test *P* = 0.04, Additional file 1: Figure S1) while 11 out 12 (92 %) Class II compounds are placed in a separate cluster based on substructure fingerprints (hypergeometric test *P* = 0.006, Additional file 1: Figure S1). Furthermore, we corroborated these observations using an independent method (multidimensional scaling, MDS) to view the pairwise distances between the compounds based on their chemical features. In the 2-dimensional MDS plot (Additional file 1: section B and Additional file 1: Figure S2), Class II compounds are enriched in a distinct quadrant of the plot (hypergeometric test *P* = 0.002, Additional file 1: Figure S2) demonstrating a strong association between their similarity in the chemical and transcriptional spaces. That is, small molecules that are chemically similar are also more likely to induce similar transcriptional responses.

**Fig. 2:**
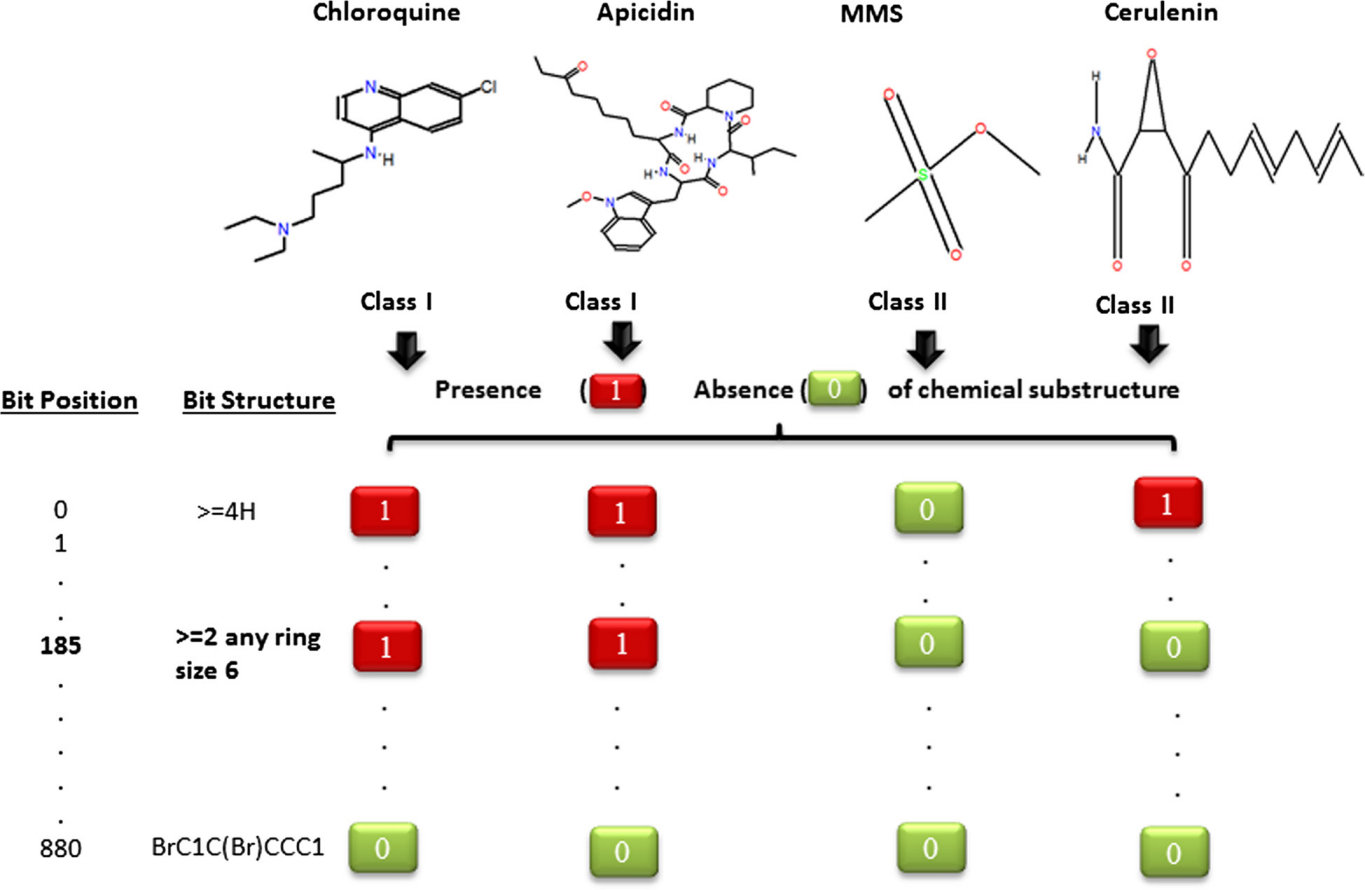
Key substructure difference between Class I and II compounds identified using the rule induction algorithm OneR. Two Class I compounds (*chloroquine and apicidin*) and two Class II compounds (*MMS and cerulenin*) are shown as an example. The substructures are numbered from 0 to 880, with the position of a given substructure referred to as its bit position. The bit structure represents a chemical element, group, ring structure or atom pairs based on the PubChem substructure fingerprints. The presence of a given substructure is encoded by 1 and the absence by a 0. Class I and II compounds are largely differentiated at bit position 185 which encodes the substructure fingerprint ‘≥2 any ring size 6’

Next, we investigated whether Class I and II compounds can be differentiated by the presence/absence of a particular chemical substructure using an unbiased approach. Application of the rule induction algorithm OneR [28, 29]) revealed that the PubChem fingerprint ‘≥2 any ring size 6’ is present in 15 out of 19 (79 %, hypergeometric test *P* = 0.01) Class I compounds as compared to only 4 out of 12 (33 %, hypergeometric test *P* = 0.002) Class II compounds (Fig. 2). This demonstrates that this substructure is enriched in Class I compounds and is associated with gene expression differences between Class I and Class II compounds.

### Drug relationships depend on both chemical structure and MOA

**Fig. 3:**
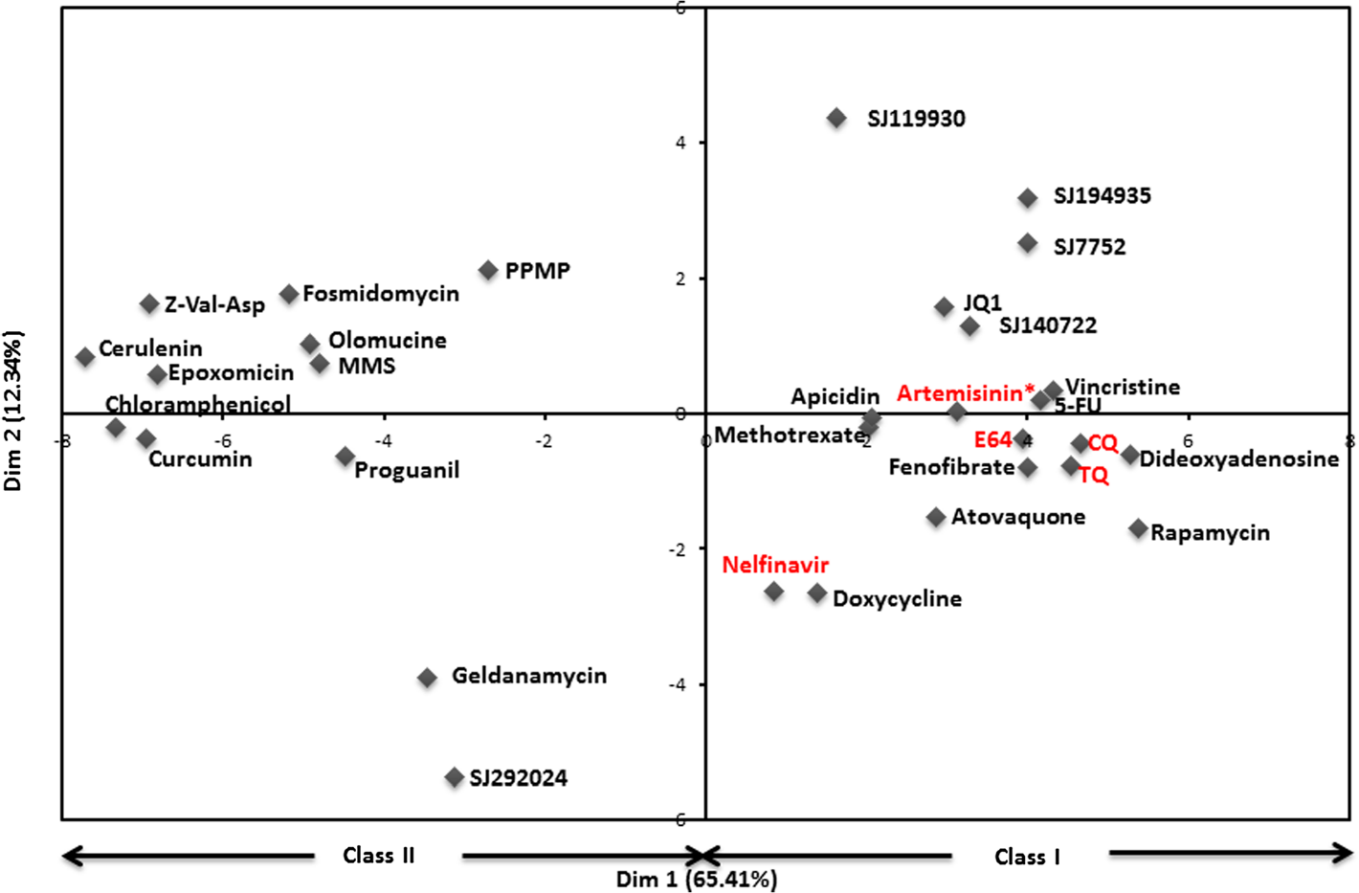
Principle component analysis (PCA) of transcriptional correlations between the small molecules. The first component of variation (Dim 1) splits the compounds into two clusters identical to those observed by hierarchical clustering of the compounds (Fig. 1). The PCA plot reveals complex drug relationships involving both chemical similarity and MOA. Compounds lacking a ring system (PPMP, olomucine, MMS, cerulenin, epoxomicin and z-Val-Asp) occupy the upper left quadrant of the plot in spite of their distinct MOAs, supporting a dominant influence of chemical structure on global small molecule relationships. An exception is E64 which occupies a position on the plot next to other hemoglobin digestion inhibitors (shown in red- CQ, TQ and E64). *Although the MOA of artemisinin is still unknown, the drug has been shown to require activation by heme released during hemoglobin digestion [56]

Correlations between small molecule transcriptional responses can arise due to two main reasons. First, small molecules that have similar chemical features are more likely to induce similar transcriptional changes [24–27]. However, not all chemically similar compounds have similar biological effects [30]. Secondly, small molecules that target the same enzyme or pathway can induce similar transcriptional changes even when their structures are unrelated [27]. To determine and quantify whether these two factors are at play in the observed small molecule relationships (Fig. 1), we performed principal component analysis (PCA) on the transcriptional correlations between the compounds (Fig. 3). We found that 65 % of the variation in correlations between the compounds is accounted for by the first dimension of variation. This dimension splits the compounds into two groups that are identical to Class I and Class II groups observed in the heat map (Fig. 1). Because these two compound groups can be largely differentiated using a single chemical group (Fig. 2), we hypothesize that chemical features account for a considerable proportion (65 %) of variation in the observed transcriptional responses, consistent with a dominant influence of chemical structure on functional similarity between drugs [27]. The aminoquinolones CQ and tafenoquine (TQ) which target hemoglobin digestion and have related chemical structures are near each other in the first two dimensions of the PCA plot (Fig. 3), supporting the hypothesis that the transcriptional responses capture both chemical similarity and MOA. However, some small molecules that differ in their structure have the same MOA. For example, E64 is chemically distinct from the aminoquinolones (CQ and TQ) but affects the same pathway (hemoglobin digestion) as this class of drugs (Fig. 3). In line with this, E64 is similar to CQ and TQ based on transcriptional effects (Fig. 3). An exception to this is nelfinavir- another hemoglobin digestion inhibitor (Fig. 3). Because the similarity between global transcriptional changes induced by the compounds does not necessarily indicate shared MOA but is heavily dependent on their chemical structure similarities [27], in the next section we develop an approach for identifying biological pathways that can be directly linked to MOA.

### Small molecules induce transcriptional responses in expected target pathways

**Fig. 4:**
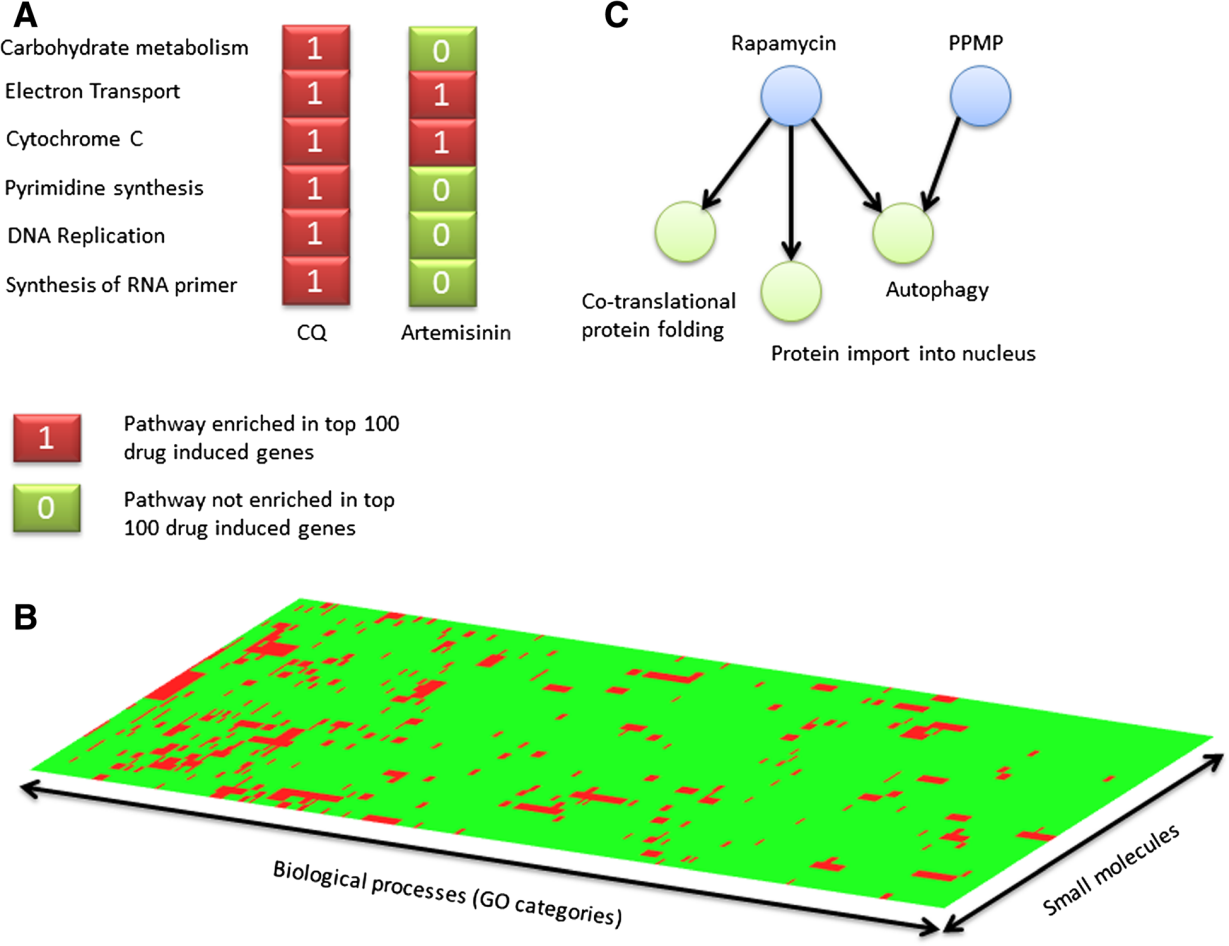
Representation of small molecule transcriptional effects as binary fingerprints in which “1” encodes an enrichment of a given biological process in the top 100 induced genes while a “0” represents lack of enrichment of a given process. **a** Two examples of partial small molecule-GO fingerprints for chloroquine and artemisnin. **b** Visualization of the small molecule–GO fingerprints for the 31 compounds as a heatmap in which the enrichment of a biological process in top 100 up-regulated genes for each compound is represented in red and the lack of enrichment by green. **c** The small molecule-GO fingerprints can be visualized in a bipartite network composed of small molecule nodes and biological process nodes. A focused view of the up-regulated small molecule-GO relationships between rapamycin and PPMP shows that both compounds are associated with autophagy but only rapamycin is associated with protein import into nucleus and co-translational protein folding

Although some small molecules exhibit high affinity towards specific protein targets, their biological effects are rarely limited to a single target and many have multiple targets [31–34]. Therefore, the effect of small molecules on biological systems can be represented in terms of how they affect a spectrum of biological processes - a profile of molecular effects rather than a single MOA. We performed biological function enrichment analysis on the top 100 up- and down-regulated genes for each small molecule perturbation (for gene lists see Additional file 4). We then constructed a binary vector for each perturbation in which each element represents whether a given biological process is enriched or not among the top 100 genes (up/down-regulated) by the small molecule (hypergeometric test *P* < 0.05; Fig. 4a and b, Additional file 5). This transformation allows small molecules’ effects to be analyzed as a bipartite network (the small molecule-GO network) consisting of two kinds of nodes: small molecules and enriched biological processes (Fig. 4c and Additional file 6). In this network, a small molecule and a biological process are connected by an edge if perturbation by that small molecule results in differential expression of genes involved in the process (hypergeometric test *P* < 0.05). This representation gives a detailed view of relationships among small molecules than is attainable with correlations or lists of GO enrichments (Fig. 4c).

In order to understand whether small molecules can induce transcriptional responses in their expected target pathways, we examined connections between each compound and biological processes in the small molecule-GO networks (Table 2 and Additional file 1: Table S1). We considered the small molecule GO-networks of up-regulated and down-regulated biological processes separately. For 19 of 25 compounds for which an expected target pathway could be established (see evidence levels in Table 1), at least one of its connections includes the target pathway (Additional file 1: Table S1). To evaluate the statistical significance of this, we asked whether each biological process was perturbed specifically only by the small molecule (s) annotated as its inhibitor (Additional file 1: Table S1). Biological processes affected by many compounds or that are highly variable across conditions cannot be reliably associated with their inhibitors, in contrast to those that are affected by only a few compounds. For example, the GO function ‘DNA strand elongation involved in DNA replication’ was enriched only in 5-FU perturbations (100 % specificity, FDR = 0), its expected inhibitor. Transcriptional responses of 8 compounds (5-FU, cerulenin, z-Val-Asp, curcumin, MMS, methotrexate, epoxomicin and E64) showed unique enrichment with their target pathways (100 % specificity, Table 2); these functions were not associated with transcriptional responses of any other compounds annotated as targeting other pathways. 72 % of small molecules with known MOA were correctly associated to their targets at a specificity threshold of 80 % (FDR = 20 %). Atovaquone- an inhibitor of the electron transport chain- resulted in down-regulation of the GO category ‘respiratory electron transport chain’ but had the lowest specificity (specificity = 61 %, Additional file 1: Table S1). This low specificity could be a consequence of the non-specific responsiveness of this pathway to unrelated chemical agents, even though only atovaquone and proguanil were expected to directly affect this pathway. The role of mitochondria in sensing stress and acting as a gateway to cell death or survival under various perturbations may also account for this [35, 36]. We provide the specificity of each small molecule association to its expected target pathway in Additional file 1: Table S1 to allow a contextual assessment of the false positive rate for each prediction in this study.

**Table 2:**
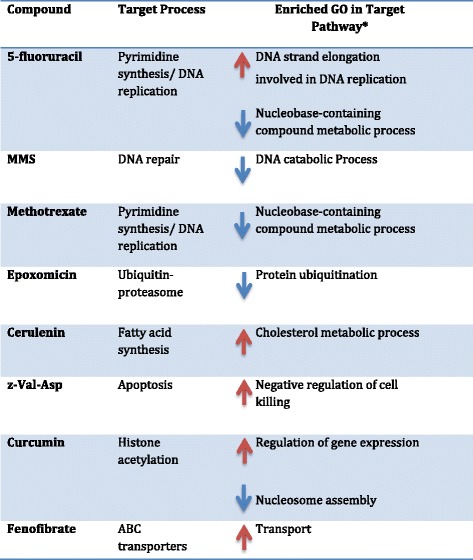
Summary of small molecule perturbations

*In ‘red arrows’ are biological processes enriched in the top 100 up-regulated genes while in ‘blue arrows’ are down-regulated biological processes

### The small molecule-GO networks predict complex relationships between drugs and biological pathways

Unlike previous work on drug-drug networks in other species [10], the small molecule-GO network provides hypotheses for why two drugs are biologically related. For example, the small molecule-GO network confirms that the drugs CQ, TQ and E64 are related through the GO process ‘proteolysis involved in cellular catabolic processes’, consistent with the effect of these drugs on hemoglobin catabolism. In contrast, curcumin and apicidin- drugs that interfere with regulation of gene expression through inhibition of histone acetylation and deactylation, respectively- are both connected to the biological process ‘regulation of gene expression’ while the drugs methotrexate and 5-FU which interfere with pyrimidine synthesis are connected to the function ‘nucleobase-containing compound metabolic process’. The connection between 5-FU and this biological function is unexpected in *P. falciparum* because the parasite lacks a pyrimidine salvage pathway which is required for the activation of 5-FU to an inhibitor of thymidylate synthase (TS) [37]. Nevertheless, 5-FU is a weak competitive inhibitor of the *P. falciparum* orotate phosphoribosyl transferase (*pfOPRT*), an essential step in pyrimidine biosynthesis [38]. Inhibition of *pfOPRT* could impact the synthesis of pyrimidines and provide a connection between methotrexate and 5-FU activity. To validate the connection between these two drugs, we measured dose responses to 5-FU across the Dd2 × HB3 genetic cross (Additional file 7) and explored potential genetic determinants of its effects through QTL. A direct *apriori* test of the genetic locus containing the *pfOPRT* gene (chromosome 5, cM 31.5) shows that parental alleles in this locus are significantly associated with 5-FU dose response with a higher IC_50_ for progeny inheriting this locus from the Dd2 parent (LOD = 1.90, *P* = 0.002). A genome-wide QTL scan demonstrates that in addition to this locus, dose response variation to the drug is associated with 3 other loci on chromosomes 8 (cM 91.8, LOD = 2.98), 11 (cM 143.2, LOD = 2.3) and 14 (cM 189.4, LOD = 1.80). The chromosome 8 QTL region includes the *Rad54* gene (PF3D7_0803400) encoding a DNA repair protein, providing additional support for the connection between 5-FU and the GO function ‘nucleobase-containing metabolic process’. Thus, the small molecule-GO network provides a framework for identifying drug similarities while at the same time identifying the biological processes underlying the similarities.

The network approach reveals that while some biological processes are widely perturbed or affected by many small molecules, others are only perturbed by only one or a few small molecules (Fig. 4c). For example, the biological categories ‘translocation of peptides into host’ (GO:0042000), ‘respiratory electron transport chain’ (GO:0022904) and ‘antigenic variation’ (GO:0020033) were up-regulated by many compounds (13, 12 and 10, respectively). These processes may reflect generalized stress responses that are associated with subsets of small molecules. In contrast, categories that are up-regulated by only one small molecule include: ‘protein import into nucleus’ (GO:0000059)- perturbed by rapamycin, ‘N-terminal protein amino acid acetylation’ (GO:0006474)- perturbed by MMS and ‘transposition’ (GO:0032196)- perturbed by 5-FU.

### Transcriptional insights into artemisinin’s MOA

**Fig. 5:**
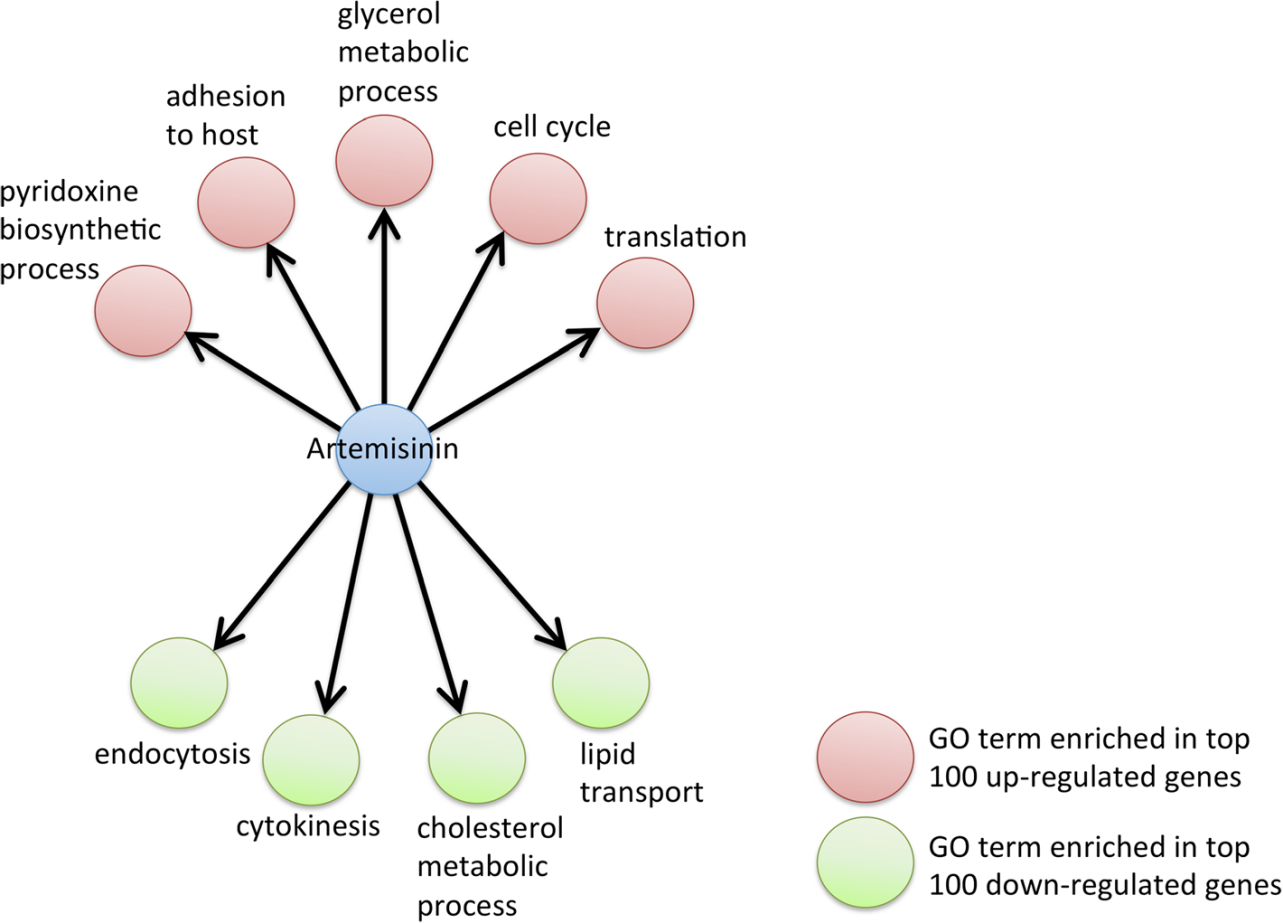
Representation of small molecule-GO network for artemisinin. GO biological processes enriched in the top 100 up-regulated genes are shown as red nodes and down-regulated genes are shown as green nodes.

The results above show that transcriptional responses can be used to predict drug MOA and provide insights into broad biological effects. The MOA of many antimalarial drugs and investigational compounds is currently unknown. Artemisinin, the front line antimalarial, has been proposed to act through the generation of free radicals leading to oxidative stress and damage of proteins [39]. However, the drug also has been associated, controversially, with direct binding to the SERCA-type Ca^2+^ ATPase protein (*pfATP6*) [40–43] and the Ca^2+^ binding translationally controlled tumor protein (TCTP) [44–48]. To mine our data for insights into artemisinin targets, we examined the small molecule-GO networks for connections involving artemisinin (Fig. 5). At 100 % specificity, the up-regulated GO connections to artemisinin are: ‘adhesion to host’ (GO:0044406), ‘glycerol metabolic process’ (GO:0006071), ‘cell cycle’ (GO:0007049), ‘translation’ (GO:0006412) and ‘pyridoxine biosynthetic process’ (GO:0008615). Recently, Mok et al. [49] identified processes in the ‘translation’ and ‘regulation of cell cycle’ GO categories as being up-regulated in parasites from Southeast Asia that have increased artemisinin clearance half-life. Furthermore, these processes were up-regulated in parasite isolates containing genetic variants associated with artemisinin resistance in the K13 gene. The reported association between artemisinin and TCTP [44–48] as well as with quiescence [50–52], is consistent with the enrichment of cell cycle function. TCTP in other organisms is involved in a variety of processes including response to stress, cell cycle, apoptosis and calcium ion binding [53–55]. In *P. falciparum*, the protein binds Ca^2+^ [45] and artemisinin [44, 46, 48]. This binding is increased in the presence of heme [48], consistent with the reported dependence between artemisinin activity and hemoglobin digestion [56]. The down-regulated GO connections to artemisinin at 100 % specificity are ‘endocytosis’ (GO:0006897), ‘cytokinesis’ (GO:0000910), ‘cholesterol metabolic process’ (GO:0008203) and ‘lipid transport’ (GO:0006869). The connection between the drug and lipid metabolism has been previously suggested [57–59] and implicated in resistance to the drug [49]. Thus, artemisinin potentially targets the cell cycle and lipid metabolism.

### Predicting MOA of novel compounds by integration of QTL

Public and private screening efforts have recently led to the identification of thousands of compounds with cytotoxic activity in *P. falciparum* [4, 5]. Techniques for predicting MOA of these compounds will be valuable for their characterization and prioritization for further development [60]. Among the compounds profiled in our study, five (SJ194935, SJ119930, SJ140722, SJ292024 and SJ77572) were derived from a high-throughput screening effort by the St Jude’s children’s hospital [5]. To ascertain the biological effects of these compounds, we determined the coherence of biological functions predicted from transcriptional perturbations with those encoded in QTL associated with dose response variation to the compounds. The small molecule-GO network demonstrates that a single compound can perturb more than one biological process. However, some functions are perturbed by a single or just a few compounds and yield predictions of higher specificity. Therefore, we prioritized the links between these compounds and each biological function by considering the specificity of each function.

Out of 13 biological processes connected to SJ194935 in the up-regulated small molecule-GO network, five (‘cell redox homeostasis’, ‘protein oligomerization’, ‘tRNA splicing’, ‘heme catabolic process’ and ‘regulation of DNA replication’) were unique to the compound. Two GO functions (‘regulation of translation’ and ‘regulation of RNA stability’) were uniquely connected to this compound in the down-regulated small molecule-GO network. These functions were evaluated for overlaps with QTL. SJ194935 dose response was associated with a genetic locus on chromosome 14 (cM 106.1, LOD = 3.12). We searched this locus for genes encoding biological processes uniquely (100 % specificity) connected to the compound in the small molecule-GO network. A biological process shared by these two approaches is ‘cell redox homeostasis’, a function that involves cytochrome genes, one of which is present in the QTL (PF3D7_1435000- cytochrome c oxidase assembly protein, putative; chromosome 14 cM 106.1, Table 3).

**Table 3:** Prediction of MOA of novel compounds from St Jude using QTL and small molecule-GO network

Small molecule	QTL peaks	LOD	Coherent functions in QTL and unique connections in network
SJ194935	14 cM 106.1	3.12	Cell redox
SJ119930	8 cM 83.2	2.18	None
SJ140722	7 cM 66.1	2.71	None
SJ292024	5 cM 65.9	2.23	Drug transmembrane transport

Similarly, to predict potential MOA of the other novel compounds, we compared the functional coherence between their unique connections in the small molecule-GO network and the functions of genes located within their QTL. For each small molecule we considered QTL peaks with at least a LOD score of 2 (Table 3; see Additional file 7 for dose responses used in QTL). The St Jude compound SJ119930 had no unique biological process connections in the up-/ down-regulated small molecule-GO networks; hence predictions could not be made at 100 % specificity. Two functions were predicted as its targets at 94 % specificity in the up-regulated network: ‘respiratory complex IV assembly’ and ‘autophagic vacuole assembly’. QTL mapping revealed a suggestive peak on chromosome 8 (cM 83.2) but we could not identify genes whose functions are coherent to those predicted in the network. For SJ140722, a single biological process (‘dolichol biosynthetic process’) was uniquely connected to it in the up-regulated network and no unique connections were observed in the down-regulated network. We did not find any gene with this function in the QTL region associated with variation in dose response to the compound. Another compound, SJ292024, was uniquely connected to the biological process ‘ribosomal large subunit biogenesis’ in the up-regulated network. Nine processes were connected to this compound at 100 % specificity in the down-regulated network: ‘protein glycosylation’, ‘respiratory chain IV complex assembly’, ‘intein mediated protein splicing’, ‘drug transmembrane transport’, ‘phosphorylation’, ‘catabolic process’, ‘DNA strand elongation involved in DNA replication’, ‘signal peptide processing’ and ‘terpenoid biosynthetic pathway’. The QTL peak associated with this compound (chromosome 5, cM 65.9) includes the gene encoding the multi-drug resistance tansporter, *pfMDR1* coherent with prediction connection between the drug and the GO function ‘drug transmembrane transport’.

## Discussion

Drug responses are complex: perturbation of a single protein can lead to direct effects on its multiple functions and indirect effects can be perpetuated through the cellular network. In addition, broad chemical properties of compounds, for example hydrophobicity, can affect multiple biological processes. Therefore, understanding the nature of transcriptional responses to specific perturbations and filtering non-specific effects requires knowledge of how cells respond to a range of chemical perturbations. Without this knowledge, it may not be possible to identify transcriptional responses specifically induced by a perturbation vs. those resulting from general stress. This may be one reason that the parasite’s transcriptional responses to perturbations have been considered to be non-specific [12, 13]. Our study design utilizes many small molecule perturbations in the same experiment and provides a novel way to disentangle transcriptional responses.

The results presented in this work demonstrate that transcriptional responses can point to drug MOA in *P. falciparum*. The observation that small molecules containing similar chemical substructures also tend to show similar transcriptional responses (Fig. 2 and Additional file 1: Figure S1) imply that these responses are specific to the perturbations. The clustering of the 31 small molecules into two broad classes (Fig. 1) coupled with the observation that a single chemical substructure (Fig. 2) can distinguish these classes implies that a large component of transcriptional responses are due to specific chemical features, most prominently rings (Fig. 2 and Additional file 1: Figure S2). Even though this study has profiled the largest set of small molecule transcriptional responses in *P. falciparum* to date, it is important to note that the 31 small molecules represent a small proportion of chemical diversity. Our study provides a foundation for more extensive studies in future.

To capture the complexity of small molecule relationships, we developed a novel framework for encoding small molecule effects into binary fingerprints of biological categories that are enriched in their perturbed gene sets (Fig. 4a, b and c). We have demonstrated that this representation allows a detailed view of complex relationships that can exist between small molecules in which some biological functions are affected by multiple compounds while others are specifically affected by a smaller number of compounds (Fig. 4c). The resulting small molecule-GO network has enabled us to tease apart small molecule-specific effects from non-specific responses, thereby providing a reliable approach for predicting small molecule MOA (Table 2 and Additional file 1: Table S1). From this network, each small molecule can be queried to determine the biological processes that are uniquely connected to it as well as those that it shares with one or more other compounds (Fig. 4c). This approach can be applied in predicting drug MOA, understanding cross-resistance between drugs, drug-drug interactions and off-target effects.

We observed that some small molecules perturb a wide array of functions, while others have a specific target pathway. Small molecules have to cross at least one membrane to reach their expected target. Broad chemical properties of small molecules such as their hydrophobicity, molecular weight, solubility and pKa could indirectly interfere with multiple biological processes. Once a small molecule reaches its target, the direct inhibition of a target enzyme, for example, can result in metabolic changes that stimulate downstream biological processes. Furthermore, drugs that bind to distinct molecular targets within the same pathway can lead to similar biological effects. It is important to identify all these levels at which a drug can have a biological effect because the therapeutic success of a drug is determined by factors such as drug transport, metabolism and resistance that in many cases are distinct from molecular targets of the drug. Most approaches for the prediction of drug MOA in *P. falciparum* rely on the identification of single targets [60]. Understanding the full spectrum of effects of a small molecule could aid in minimizing off-target effects and enhance the design of highly specific drugs.

Our study uncovers some potential challenges of using transcriptional perturbations for predicting drug MOA and reveals limitations that will need to be addressed in future studies. First, a large library of perturbations is needed in order to filter non-specific effects of small molecules. Secondly, predicting the MOA of small molecules that target biological processes that are non-specifically associated with numerous perturbations will pose a bigger challenge than those of small molecules that affect pathways that respond to only very few compounds. For example, the biological process “respiratory electron transport chain” was connected to 12 compounds in the small molecule-GO network, increasing the FDR for predicting the MOA of compounds affecting this process. However, as more compounds are profiled, the FDR for such compounds could be reduced. The small molecule-GO network provides a way to identify these non-specific effects and is generalizable for the prediction of drug MOA in other organisms.

## Conclusions

We have shown the utility of transcriptional perturbations in predicting drug MOA and provide novel biological insights. Similarities between transcriptional responses of small molecules depend on their chemical composition and MOA. In particular, small molecules containing similar chemical constituents induce similar transcriptional responses. Furthermore, we determine that the presence/absence of rings in a compound dominate transcriptional similarities between compounds. We further show that small molecules with the same MOA induce similar transcriptional responses. Using a novel network representation (the ‘small molecule-GO network’), we demonstrate that some biological processes are affected by many compounds, potentially reflecting generalized stress responses, while others are affected by only one or few compounds. This network approach provides a hypothesis-testing framework for explaining similarities between any two or more small molecules based on the biological processes to which they are connected in the network. The small molecule-GO network correctly identifies the MOA of 72 % of compounds at a specificity of 80 %, establishing that this approach can be applied for predicting MOA. The representation of transcriptional responses as a small molecule-GO network is a novel approach that can be generalizable to other organisms.

## Methods

### Parasite cultures

Parasite clones Dd2 and HB3 were cultured using standard methods in human red blood cells (Indiana Regional Blood Center, Indianapolis, Indiana) suspended in complete medium (CM) containing RPMI 1640 with L-glutamine (Invitrogen Corp.), 50 mg/L hypoxanthine (Sigma-Aldrich), 25 mM HEPES (Cal Biochem), 0.5 % Albumax II (Invitrogen Corp.), 10 mg/L gentamicin (Invitrogen Corp.) and 0.225 % NaHCO3 (Biosource) at 5 % hematocrit. Cultures were grown separately in sealed flasks at 37 °C under an atmosphere of 5 % CO_2_, 5 % O_2_, and 90 % N_2_. Small molecules were purchased from Sigma-Aldrich with the exception of JQ1 (provided by Dr. James Bradner, Harvard Medical School) and the compounds SJ194935, SJ119930, SJ77572, SJ292024 and SJ140722 (provided by Dr. Kiplin Guy, St Jude’s Children’s hospital). Sorbitol double-synchronized cultures of the two clones were exposed briefly for 2 h to each compound at a single developmental stage (24 h trophozoites) at a concentration (IC_50_) obtained from literature sources or determined by our laboratory (Table 1).

### RNA extraction and cDNA synthesis

Total RNA was extracted from 20mls of culture using TriZol reagent (Invitrogen, Carlsbad, CA) as described previously [61]. Quantity of RNA and protein/organic contamination were determined using Nanodrop (NanoDrop Technologies). 300 ng of RNA was used as starting material for cDNA synthesis using the Sigma WTA2 whole transcriptome amplification kit (Sigma Aldrich, St Louis, MO). The cDNA synthesis reaction was performed in two steps: library synthesis and library amplification. To synthesize the cDNA library, 300 ng of sample RNA was incubated with reverse transcriptase and non-self-complimentary primers that contain a quasi-random 3’ end and a universal 5’ end. Primer extension was then performed using WTA2 polymerase to generate an OmniPlex cDNA library consisting of random, overlapping 100 to 1000 base fragments flanked by a universal end sequence. Amplification was then performed using primers targeting the universal 5’ ends. cDNA cleanup was performed using 3 M sodium acetate and ethanol.

1 μg of cDNA was labeled with Cy3 dye using 65 % AT rich pre-labeled random hexamers as primers for cDNA synthesis by Klenow fragment of DNA polymerase I. All samples were hybridized to a custom Nimblegen 12-plex microarray containing 128,179 probes, approximately 22 probes per annotated gene (PlasmoDB v6.3) with an average of 5 probes per exon (see Additional file 1: section C and Additional file 1: Figure S3 for microarray validation information). Hybridizations were performed for 22 h followed by washing of the arrays as described according to standard protocols (Roche NimbleGen Inc., Madison, WI). The microarray image was obtained using a 2uM scanner and probe intensity values extracted using NimbleScan software (Roche NimbleGen Inc., Madison, WI).

### Microarray data processing

Probe intensities were normalized using robust multichip average (RMA) method [62]. This normalization was performed across all samples hybridized on a single chip. Transcript level for each gene was obtained by averaging the processed signal intensity of all the probes across its exons as follows. Exon signal intensity for each gene was obtained by averaging the intensities of all probes within each exon. To determine a significance threshold for exon expression levels, a background distribution of signal intensities from a set of 10,000 negative control probes with no sequence matches to the *P. falciparum* genome was generated. A threshold corresponding to the 95^th^ percentile (5 % FDR) of the signal distribution of the negative control probes was then applied [63]. To determine gene expression levels, exons that passed the 5 % significance threshold were subjected to an additional threshold derived from intensities of 1000 simulated exons each consisting of 20 randomly sampled negative control probes. Intensities of exons that passed a 5 % FDR threshold based on this background distribution were averaged to obtain an average transcript level for each gene.

### Analysis of pairwise relationships between compounds using genome-wide response profiles

For each perturbation, we averaged the signal intensity for each gene across two lab clones (Dd2 and HB3). The resulting data was then used to obtain a compound-specific response index for each gene by normalizing the gene’s average transcript level following perturbations in the two clones against its average level across all perturbations within the same experimental batch to obtain a gene specific response index. The global transcriptional response to a small molecule was then represented as a vector where each element represents a gene specific response index. This vector is referred to as the genome-wide response index. Small molecule global transcriptional relationships were determined using Pearson correlations between their genome-wide response indices. Clustering and visualization of the correlation matrix was performed in R. To determine the components of variation in the global correlations between small molecule responses, PCA was performed in R using the PCA function in the package FactoMineR.

### Analysis of small molecule relationships based on chemical fingerprints

Small molecule relationships in the chemical space were determined by first converting each small molecule into a binary fingerprint of 881 elements using the PubChem 2-dimensional substructure fingerprints [64]. Each position in the binary fingerprint encodes the presence/absence of a substructure such as a specific chemical element, a type of ring system, atom pairing, or atom environment, etc. A full description of the fingerprints can be found at [65].

We downloaded chemical structure files (SDF) for each small molecule from PubChem. Transformation of chemical structures into substructure fingerprints was performed in the R statistical package ChemmineR [66]. Small molecule relationships based on the fingerprints were then visualized by hierarchical clustering. To determine whether chemical structure accounts for the observed major clustering of small molecules into two main clusters (named Class I and Class II), small molecules were projected onto a two-dimensional surface using multidimensional scaling (MDS) in the MASS package [67] of the R statistical software. Chemical substructures that discriminate Class I from Class II compounds were identified using a rule induction algorithm (OneR) in the WEKA machine learning package [28]. The OneR algorithm produces a single ‘If-Then’ rule that identifies a single predictor variable that differentiates between two outcomes [29]. The ability of the rule to discriminate Class I and II compounds was evaluated by testing the enrichment of Class I and II molecules when the rule is applied to the substructure fingerprints of all the small molecules. The enrichment *P*-values were obtained by hypergeometric tests.

### Encoding small molecule effects into binary biological process fingerprints

GO enrichment analysis was performed on the most responsive genes in the transcriptome (the top 100 [~2 %] most up- and down-regulated genes) and enriched biological processes (hypergeometric test FDR corrected *P* < 0.05) were determined using the online platform MADIBA [68]. For each small molecule, we then represented the transcriptional response as a binary vector whose elements are biological process categories that have a value of 1 (when the process is enriched following the perturbation) and 0 (when the process is not enriched).

### Construction of small molecule-GO networks

To determine the relationships between small molecules to specific biological processes, we constructed small molecule-GO bipartite networks in which nodes were either small molecules or biological processes. Small molecules were connected by an edge to a biological process if perturbations by the small molecule were associated with an enrichment (hypregeometric test *P* < 0.05) of the biological process in the most responsive genes to the perturbation (top 100 up- and down-regulated genes).

### Analysis of the effect of small molecules on their expected target pathways

The effect of small molecules on their expected target pathways was determined by querying the small molecule-GO network in the network visualization software Cytoscape [69]. The expected target pathway for each small molecule was determined from literature and the enzyme database BRENDA. For each small molecule, we determined whether the biological processes it is connected to in the small molecule-GO network includes its expected target pathway. An estimate of the FDR for the association between a small molecule and its expected biological pathway was computed as the proportion of small molecules that were also connected to that category in the network. A small molecule was regarded as specifically connected to its expected target pathway if the FDR was less or equal than 10 %.

### Dose response assays and QTL

We performed standard dose response assays across the Dd2 × HB3 recombinant clones cultured under varying concentrations of the drugs 5-FU, methotrexate, SJ194935, SJ119930, SJ140722 and SJ292024. Dose response assays were performed as previously described [70] using 4 to 6 replicates of parasite cultures of 37 recombinant clones of the Dd2 × HB3 genetic cross, including the parental lines. Quantitative trait locus (QTL) analysis for the dose responses was performed using previously published statistical methods in Pseudomarker v 2.04 [71] and the Dd2 × HB3 genetic cross microsatellite linkage maps. The statistical significance of the obtained log odds scores (LOD) were obtained from a chi-square distribution, *P* = 1 - chi2cdf (2 × LOD score × Log_10_, degree of freedom = 1) where chi2cdf is the Matlab chi-square cumulative distribution function. Gene candidates were considered as those lying within the region bounded by the physical location of the genetic marker reported with the highest LOD score and the nearest markers in the region.

### Prediction of MOA of novel compounds

The MOAs of five novel compounds (SJ194935, SJ119930, SJ140722, SJ292024 and SJ77572) emerging from a recent phenotypic screen [5] were predicted by combining predictions from the small molecule GO network and QTL analysis. To predict MOA from the small molecule-GO network, the network was queried in Cytoscape [69] and biological process connections that are uniquely connected to a given small molecule of interest selected as potential targets. Separately, dose response assays were determined for each of the compounds in 37 parasite lines in the Dd2 × HB3 genetic cross followed by QTL analysis. Gene candidates for each small molecule at a given QTL locus was then determined as genes located upstream and downstream of the region lying between the physical location of the QTL marker and the nearest marker.

The gene candidates at the QTL for a given novel compound were then examined for any functions that are related to those predicted as uniquely connected to the compound in the small molecule-GO network. The functions shared between the candidate genes from QTL and those from the small molecule-GO network were then regarded as potential targets of the compound.

For arteminsin, the functions shared between its unique biological process connections in the small molecule-GO network and previous reports were considered as its MOA [50–52].

## Availability of supporting data

The raw data set supporting the results of this article is available in the NCBI Gene Expression Omnibus repository, [GEO:GSE67127, http://www.ncbi.nlm.nih.gov/geo/query/acc.cgi?acc=GSE67127]. The processed data sets supporting the results of this article are included within the article (and its additional file (s)).

## Additional files

Additional file 1:
**Supplementary text and figures.** (DOCX 441 kb) Additional file 2:
**Gene Expression Data.** (XLSX 6956 kb) Additional file 3:
**PubChem Fingerprints.** (XLSX 98 kb) Additional file 4:
**Top 100 and Bottom 100 gene lists for each drug perturbation.** (XLSX 487 kb) Additional file 5:
**Drug GO-Matrix.** (XLSX 58 kb) Additional file 6:
**Small Molecule GO-networks.** (XLSX 30 kb) Additional file 7:
**Dose Response Profiling.** (XLSX 15 kb)
