# Supplementary material for: An integrative analysis of small molecule transcriptional responses in the human malaria parasite Plasmodium falciparum
Source: BMC Genomics. 2015 Dec 4;16:1030. doi: 10.1186/s12864-015-2165-1 (PMC4670519; doi:10.1186/s12864-015-2165-1)
Supplement: Additional file 1: — Supplementary text and figures. (DOCX 441 kb) [file 12864_2015_2165_MOESM1_ESM.docx]

**Additional file 2: Supplementary text and figures**

1. **Chemical Relationships between small molecules**


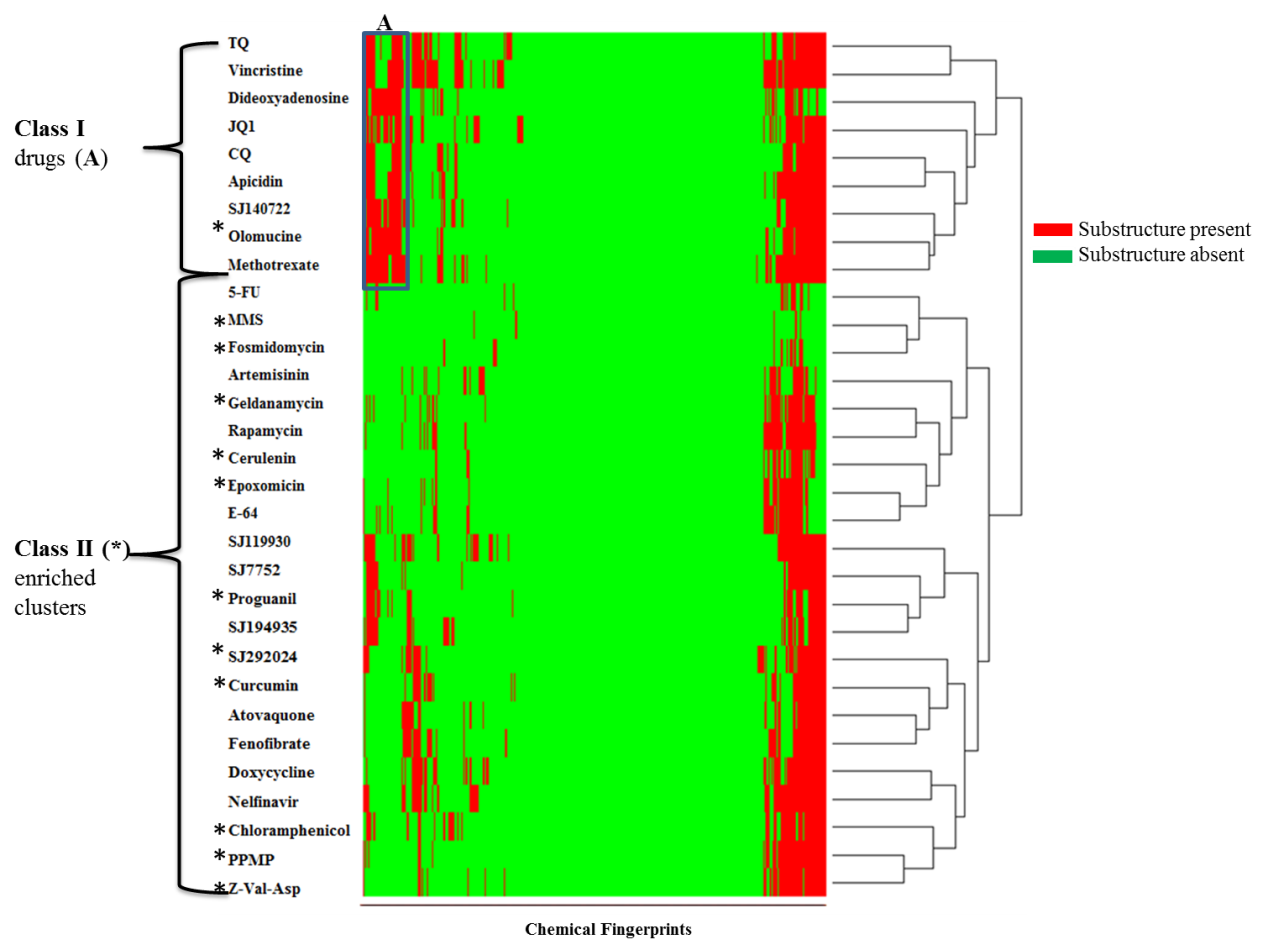


**Chemical fingerprints**

**Class I enriched cluster**

**Class II enriched cluster**

**Tafenoquine**

**Vincristine**

**Dideoxyadenosine**

**JQ1**

**Chloroquine**

**Apicidin**

**SJ140722**

**Olomucine**

**Methotrexate**

**5-Fluorouracil**

**MMS**

**Fosmidomycin**

**Artemisinin**

**Geldanamycin**

**Rapamycin**

**Cerulenin**

**Epoxomicin**

**E64**

**SJ119930**

**SJ7752**

**Proguanil**

**SJ194935**

**SJ292024**

**Curcumin**

**Atovaquone**

**Fenofibrate**

**Doxycycline**

**Nelfinavir**

**Chloramphenicol**

**PPMP**

**z-Val-Asp**

**Figure S1:** Clustering of small molecules based on their PubChem substructure fingerprints. We designate Class I drugs as those transcriptional responses (Figure S1) that are enriched for one substructure fingerprint cluster (hypergeometric test *P* = 0.04) and share a combination of substructures highlighted by (A). Class II drugs are enriched in a different cluster based on substructure fingerprints (hypergeometric test *P* = 0.006).

1. **Relationships among Class I and II small molecules in a 2-dimensional space**

To explore the relationship between chemical composition and transcriptional responses, we applied multidimensional scaling (MDS) to the substructure fingerprints and projected the compounds onto a two-dimensional surface (Additional file 2: Figure S2). MDS is a mathematical technique for dimensionality reduction in which objects in a high dimensional space can be compared in a low dimensional space in which the distances between the objects is determined by their similarity or dissimilarity across the dimensions. We applied MDS because it allows substructure relationships between pairs of the small molecules to be assigned distances on each dimension, in which the 881 substructures are reduced into two coordinates of variation similar to principal component analysis (PCA). Application of the method to the small molecule substructures fingerprints shows that Class I compounds (red) are enriched in two regions of the resulting two-dimensional surface (Additional file 2: Figure S2, regions A and B) while Class II compounds (green) are concentrated on a different region (region D). Class I compounds in region A and B contain at least two rings in contrast to Class II compounds which are enriched in region C and contain at most one ring. 17 of 19 (89%) Class I compounds contain at least 2 rings compared to 4 of 12 (33%) of Class II compounds.


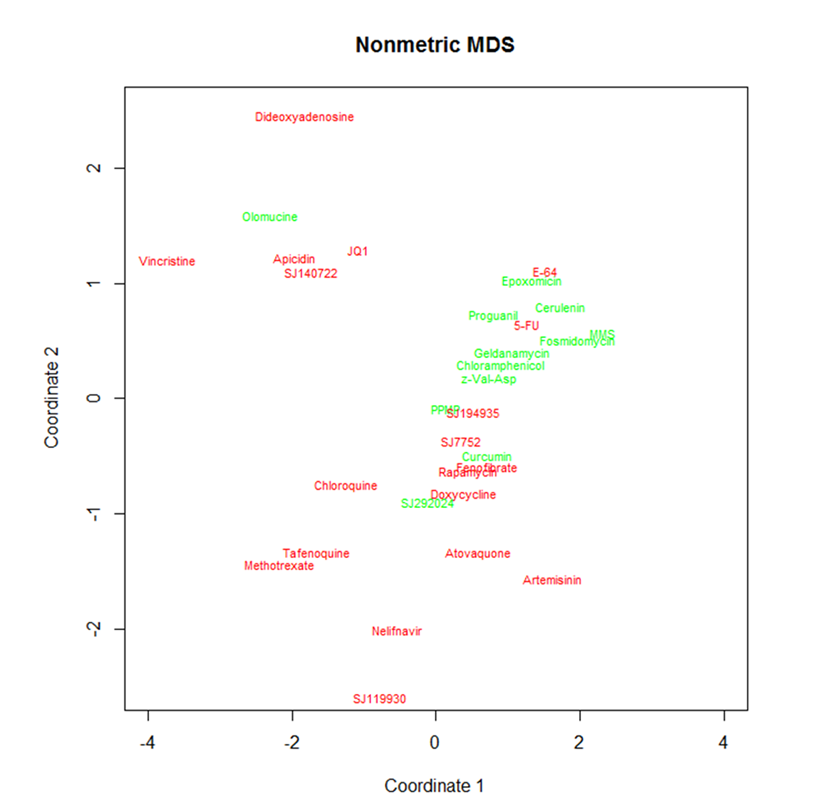


Region B

Region C

Region A

**Figure S2:** Visualization of small molecules relationships on a two-dimensional MDS plot comparing their pairwise distances based on the chemical substructures they contain. Two-dimensional MDS was performed to test whether broad clustering of small molecules based on transcriptional responses (Figure 1) reflects an underlying existence of two components of variation in chemical fingerprints. Class I (red) compounds cluster together in regions A and B, while Class II compounds are enriched in region C. The placement of Class I and II compounds in different regions on the plot indicates that underlying chemical substructure relationships are related to the transcriptional small molecule relationships.

**Table S1: Specificity of biological process enrichments in the top 100 up- and down-regulated gene sets**

| Compound | Target Process |  | Enriched GO in Target Pathway |  | Specificity (1-FDR) |
| --- | --- | --- | --- | --- | --- |
| 5-fluoruracil | Pyrimidine synthesis/ DNA replication |  | DNA strand elongation involved in DNA replication  Nucleobase-containing compound metabolic process |  | 100%  100%* |
| Proguanil | Mitochondrial electron transport |  | Mitochondrial electron transport |  | 87%  87% |
| MMS | DNA repair |  | DNA catabolic process |  | 100% |
| Doxycycline | Organellar translation |  | Tyrosyl tRNA aminoacylation |  | 97% |
| Atovaquone | Mitochondrial electron transport |  | Respiratory electron transport chain |  | 68%*  61%* |
| Chloroquine | Hemoglobin metabolism |  | Proteolysis involved in cellular catabolic process |  | 84%* |
| Chloramphenicol | Organellar translation |  | Translational elongation |  | 90% |
| Tafenoquine | Hemoglobin metabolism |  | Proteolysis involved in cellular catabolic process |  | 84% |
| Methotrexate | Pyrimidine synthesis/ DNA replication |  | Nucleobase-containing compound metabolic process |  | 100% |
| Rapamycin | Autophagy |  | Autophagy |  | 97% |
| E64 | Hemoglobin metabolism |  | Proteolysis |  | 90% |
| Epoxomicin | Ubiquitin-proteasome |  | Protein ubiquitination |  | 100% |
| Cerulenin | Fatty acid synthesis |  | Cholesterol metabolic process |  | 100% |
| z-Val-Asp | Apoptosis |  | Negative regulation of cell killing |  | 100% |
| Apicidin | Histone  deaceytlation |  | Regulation of transcription |  | 90% |
| Dideoxyadenosine | Signaling |  | Signal transduction  Intracellular receptor mediated signaling |  | 94%*  81% |
| Curcumin | Histone acetylation |  | Regulation of gene expression  Nucleosome assembly |  | 100%  100% |
| Olomucine | Phosphorylation |  | Deoxyribonucleoside diphosphate metabolic process; Signal transduction |  | 94% |
| Fenofibrate | ABC transporters |  | Transport  Plasma membrane ATP synthesis coupled proton transport |  | 100%  74% |

The expected target pathways of the following drugs were not enriched in the top 100 up- or down-regulated genes and are excluded from this table: geldanamycin, nelfinavir, vincristine, fosmidomycin and PPMP.

*The specificity takes into account the possibility that some small molecules target the same GO functions. For example, 5-FU and methotrexate both affect pyrimidine synthesis/ DNA replication and are therefore associated with the GO function nucleobase-containing compound metabolic process; chloroquine, tafenoquine and E64 inhibit hemoglobin proteolysis. In ‘red arrows’ are biological processes enriched in the top 100 up-regulated genes while in ‘blue arrows’ are down-regulated biological processes.

Specificity = Number of small molecules that lead to up/ down-regulated of the GO process

Number of small molecules annotated as affecting GO processes in the pathway

**C. Nimblegen Microarray Description and Validation**

Our custom 12-plex Nimblegen microarray contains 128,179 probes of 50 to 75-mers representing 5,683 annotated transcripts in PlasmoDB (v. 6.3). Each transcript is represented by ~22 probes, with an average of 5 per exon. In addition, 10,000 negative control probes, having no sequence similarity to the *P. falciparum* genome but of the same GC content and melting temperature as the transcript probes, were randomly generated and placed on the array.

To validate the Nimblegen exon array we compared biological reps of HB3 at 4 timepoints (12, 24, 36 and 48 hpi) using our custom Nimblegen array and a previously published Agilent 8×15K array [1]. The correlation between the genome-wide log_2_ ratios of the 36 versus 12 hour samples hybridized on the Nimblegen exon array and the previously published Agilent array (r = 0.85) across 4464 genes for which expression was detected in both arrays is comparable to that observed between technical replicates of the Agilent array [1]. Furthermore, we used GSEA to check concordance in up-regulated genes between the Nimblegen and Agilent platforms. To perform GSEA, we constructed a gene set composed of the up-regulated genes on the Agilent array followed by the examination of the list of genes sorted by their level of decreasing expression in 36 vs 12 hr on the exon array. The top part of this list was significantly enriched with genes up-regulated in the Agilent array (Figure S3 A, GSEA *P < 0.001*), demonstrating a high concordance in biological information between the 2 platforms. For example, in the top 20 up-regulated genes on the exon array, 19 (95%) were up-regulated on the Agilent array and 20 (100%) were up-regulated in the DeRisi transcriptome [2] (Figure S3 B); in the top 100 genes, the agreement to the Agilent array was 92% and in the top 500 the agreement to the Agilent array was 77%.


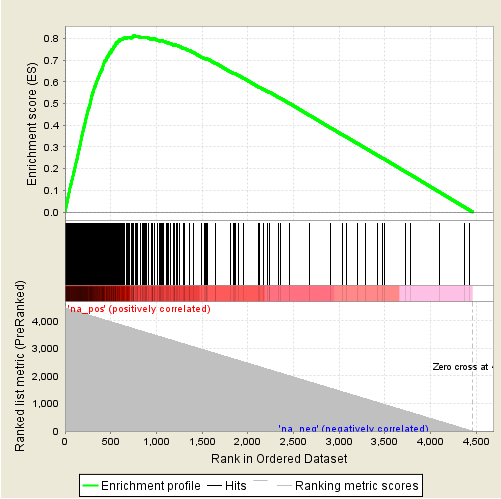


B

A


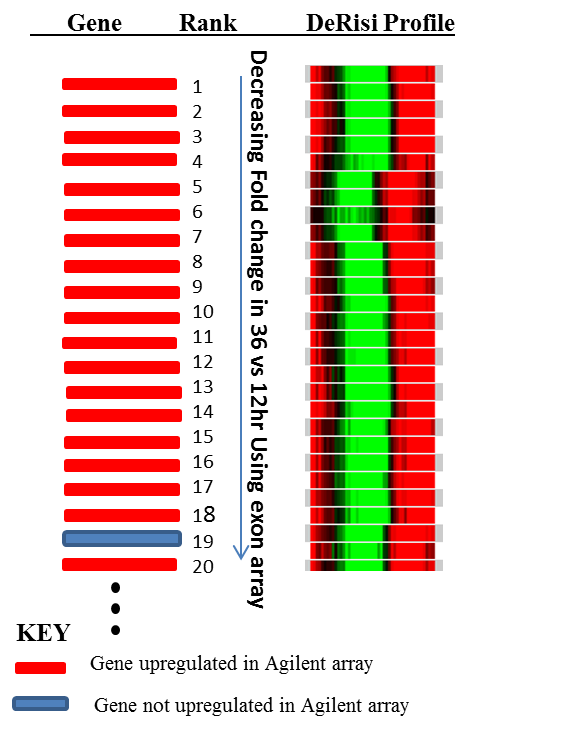


Timepoint (hrs)

0 24 48

Figure S3. Application of gene set enrichment analysis (GSEA) to compare Nimblegen and Agilent platforms in a threshold robust manner. (A) GSEA plot showing the distribution of up-regulated genes detected on the Agilent array (vertical black lines) in a list of genes sorted by their fold change in 36 vs 12 hr on the exon array. Most of the up-regulated genes reported from the Agilent array are present in the top part of the ranked list obtained from the exon array showing that the two platforms are highly concordant (GSEA *P* < 0.001). Each black vertical line beneath the green plot indicates an up-regulated gene in the Agilent array. The position of the vertical line reflects its fold change in 36 vs 12 hr in the exon array with the left most vertical line representing the most up-regulated gene in this array. The fold change of each gene in the exon array decreases as you move from left to right on the plot. The high density of black vertical lines in the left most part of the plot indicates that up-regulated genes in the Agilent array have high fold change in the exon array. (B) Graphical depiction of the top 20 up-regulated genes from the exon array. Genes are colored in red if they are reported as up-regulated on the Agilent array and blue if they are not considered as up-regulated by the Agilent array. The DeRisi profile shows the relative expression levels of the same genes (numbered 1 to 20) across the 48 hr life cycle based on the DeRisi Lab Malaria Transcriptome Database. The DeRisi profiles of the genes across the cycle are colored red if they are up-regulated at that particular time point and green if they are down-regulated. The DeRisi profiles demonstrate that the top 20 most up-regulated genes on the exon array are also up-regulated based on the DeRisi data, including gene number 19 which is down-regulated on the Agilent array but up-regulated on the exon array.

**D. Averaging perturbation responses across strains identifies drug MOA reliably**

Gene expression response to perturbations can vary due to strain-specific differences in response to stress and/ or growth inhibition due to underlying genetic differences between strains and regulatory variation across parasite lines as previously reported in our publication on multiple expression QTL (eQTL) [3] in a *P. falciparum* genetic cross. Observations from only a single strain may not therefore be adequate to determine MOA. Combining data from 2 or more strains can help filter out non-specific drug responses, as these responses are more likely to vary among strains that drug specific responses which are associated with specific pathways. In this study we obtained gene expression data from 2 different drug perturbed clones (HB3 and Dd2) as biological replicates. Since these 2 clones are totally independent, it can be expected that any transcriptional responses that they share in common to a single drug but not others reflect drug specific responses and not generalized stress response. Previous transcriptional perturbations in *P. falciparum* appeared to detect only generalized stress response, however, those experiments were performed in a single strain and responses were only determined for a single drug, making it very difficult to separate drug specific and general stress responses [4, 5]. We hypothesized that if the transcriptional responses are non-specific, then similar biological functions would be enriched in the induced genes across multiple drugs. Conversely, drug specific responses would be signified by the unique overlap of specific functional categories between biological replicates of a given drug.

We found that GO functional categories overlapping between biological replicates of each drug were highly specific to the drug and were not observed as overlapping for replicates of other drugs in a pilot study of 10 drugs (Table below). Only 2 biological categories were shared between replicates of more than one drug (Table highlighted in yellow). The overlapping/ shared biological functions between replicates for a given drug can therefore be considered as drug specific responses.

To determine whether the drug specific response for each drug constitute a large proportion of the observed response, we computed a P-value measuring the significance of the observed number of overlapping GO Biological Process categories between replicates of each drug, given the total number of categories in the Biological Process hierarchy of *P. falciparum*. A low P-value (P < 0.05) indicates that the drug specific response constitutes a large proportion of the transcriptional response while a high P-value indicates the reverse. One caveat of this P-value is that some genes participate in multiple biological processes and are thereby annotated in multiple GO categories. Such genes skew the magnitude of overlaps. The only drugs for which the drug specific response constituted a large proportion of the observed transcriptional response were 5-FU (*P* = 6.5 x 10^-11^), Proguanil (*P* = 4.4 x 10^-7^) and Doxycycline (*P* = 0.0002).

Because the specific drug response of some drugs does not constitute a large proportion of the observed response in a single strain, this implies that performing replicates across strains is critical for filtering out the strain specific generalized transcriptional responses. For example, for the drug artemisinin, we observed multiple GO categories as enriched in each strain (Dd2- 23 categories, HB3- 20 categories), but only one GO category was shared. This GO category (GO:0006414, translational elongation) is however highly specific because it is not shared by any of the other replicates of other drugs. It is also a biological function that was observed to be enriched in overexpressed genes in artemisinin resistant parasites [6]. Using data from 2 strains not only filters the data, but also helps refine a more restricted mechanism of action that is amenable to experimentation. While the GO categories that are not shared between replicates of a drug perturbation may be important in modulating the drug response, they are likely to be less fundamental for the drug MOA and are beyond the current scope. Understanding such responses would require replicates within each strain. In our subsequent analyses, we therefore use the average transcriptional response from the 2 strains.

| **Drug** | **Shared GO categories** | **Are categories specifically shared in replicates of one drug only?** | **P-value of Overlap** |
| --- | --- | --- | --- |
| Chloroquine | "GO:0006118" "GO:0051603" "GO:0031338" | Yes | 0.05 |
| Artemisinin | "GO:0006414" | Yes | 0.3 |
| Atovaquone | "GO:0015986" "GO:0006457" | No, Shared with TQ | 0.09 |
| 5-FU | "GO:0016575" "GO:0018279" "GO:0007035" "GO:0002377" "GO:0006829" "GO:0009052" "GO:0050776" "GO:0006437" "GO:0007076" "GO:0032196" "GO:0044237" "GO:0006269" "GO:0006271" | "GO:0006437" shared with Doxycycline | 6.5 x 10^-11^ |
| Proguanil | "GO:0020033" "GO:0042000" "GO:0009405" "GO:0006986" "GO:0009408" | Yes | 4.4 x 10^-7^ |
| MMS | "GO:0030522" "GO:0046907" | Yes | 0.1 |
| Doxycycline | "GO:0006437" "GO:0007018" "GO:0007017" "GO:0006633" | Yes | 0.0002 |
| Tafenoquine | "GO:0006428" "GO:0015986" "GO:0007264" | No, Shared with Atovaquone | 0.07 |
| Methotrexate | None | N/A | N/A |
| Chloramphenicol | "GO:0006099" | Yes | 0.3 |

References

1. Kafsack BF, Painter HJ, Llinas M: **New Agilent platform DNA microarrays for transcriptome analysis of Plasmodium falciparum and Plasmodium berghei for the malaria research community.** Malar J 2012, **11**:187-2875-11-187.

2. Llinas M, Bozdech Z, Wong ED, Adai AT, DeRisi JL: **Comparative whole genome transcriptome analysis of three Plasmodium falciparum strains.** Nucleic Acids Res 2006, **34**(4):1166-1173.

3. Gonzales JM, Patel JJ, Ponmee N, Jiang L, Tan A, Maher SP, Wuchty S, Rathod PK, Ferdig MT: **Regulatory hotspots in the malaria parasite genome dictate transcriptional variation.** PLoS Biol 2008, **6**(9):e238.

4. Ganesan K, Ponmee N, Jiang L, Fowble JW, White J, Kamchonwongpaisan S, Yuthavong Y, Wilairat P, Rathod PK: **A genetically hard-wired metabolic transcriptome in Plasmodium falciparum fails to mount protective responses to lethal antifolates.** PLoS Pathog 2008, **4**(11).

5. Gunasekera AM, Myrick A, Le Roch K, Winzeler E, Wirth DF: **Plasmodium falciparum: genome wide perturbations in transcript profiles among mixed stage cultures after chloroquine treatment.** Exp Parasitol 2007, **117**(1):87-92.

6. Mok S, Imwong M, Mackinnon MJ, Sim J, Ramadoss R, Yi P, Mayxay M, Chotivanich K, Liong KY, Russell B, Socheat D, Newton PN, Day NP, White NJ, Preiser PR, Nosten F, Dondorp AM, Bozdech Z: **Artemisinin resistance in Plasmodium falciparum is associated with an altered temporal pattern of transcription.** BMC Genomics 2011, **12**:391-391.
